# Pre-aspiration outpatient ultrasound can accurately predict dry taps in prosthetic hips suspected of infection; a prospective study

**DOI:** 10.3389/fsurg.2024.1410465

**Published:** 2024-08-30

**Authors:** Alireza Mirzaei, Seyyed Hossein Shafiei, Mohsen Sadeghi-Naini, Masih Rikhtehgar, Mansour Abolghasemian

**Affiliations:** ^1^ Bone and Joint Reconstruction Research Center, Department of Orthopedics, School of Medicine, Iran University of Medical Sciences, Tehran, Iran; ^2^ Department of Orthopedic Surgery, University of Minnesota, Minneapolis, MN, United States; ^3^ Orthopedic Surgery Research Center, Sina University Hospital, Tehran University of Medical Sciences, Tehran, Iran; ^4^ Department of Neurosurgery, Faculty of Medicine, Lorestan University of Medical Sciences, Khoram Abad, Iran; ^5^ Department of Surgery, Faculty of Medicine and Dentistry, University of Alberta, Edmonton, AB, Canada; ^6^ Division of Orthopedics, Moheb-Mehr Hospital, Tehran, Iran

**Keywords:** prosthetic joint infection, dry tap, hip aspiration, ultrasound

## Abstract

**Introduction:**

Aspiration represents the most potent method for exploring the potential occurrence of Periprosthetic Joint Infection (PJI). However, dry taps are common. While aspiration under ultrasound (US) guidance in the radiology department has become increasingly popular, hip aspiration is still routinely conducted in the operating room (OR) under x-ray guidance in numerous medical centers. When conducted within the confines of the OR, a dry tap aspiration not only subjects the patient to an unnecessary invasive procedure but also constitutes a substantial strain on OR time and resources. Our objective was to assess whether an outpatient US conducted before aspiration could reliably predict the likelihood of encountering a dry hip aspiration.

**Methods:**

In a prospective study, we enrolled 50 hips who were suspected of PJI and slated for revision total hip arthroplasty and required hip aspiration. Before the aspiration procedure, we conducted an outpatient hip ultrasound (US) to assess the presence of fluid collection. Subsequently, all patients underwent aspiration under fluoroscopy in the OR, irrespective of the ultrasound findings We then assessed the level of agreement between the ultrasound results and the outcomes of hip aspiration.

**Results:**

The US exhibited a sensitivity of 95.7% (95% CI 69.8–91.8), a specificity of 74.1% (95% CI 52.8–91.8), a positive predictive value of 75.9% (95% CI 50.9–91.3), and a negative predictive value of 95.2% (95% CI 71.3-99.8) in predicting the success of aspiration.

**Discussion:**

Pre-aspiration outpatient US demonstrates a high degree of accuracy in predicting dry taps in these patients. We recommend its incorporation into the hip aspiration procedure in medical centers where aspiration is performed in the operating room. In the broader context, these findings reinforce the preference for US-guided aspiration within the radiology department over x-ray-guided aspiration in the operating room since about ¼ of the positive USs for hip collection will lead to a dry tap if the aspiration is performed in the OR under fluoroscopy guidance.

## Introduction

Total hip arthroplasty (THA) is a reliable procedure for the treatment of end-stage hip pathologies such as osteoarthritis that provides excellent clinical outcomes and high satisfaction levels (1). The rate of THA is steadily increasing due to the aging population (2) and the growing trend of using this treatment in younger patients (3). In tandem with the increasing number of THAs, revision rates have steadily grown (4).

Periprosthetic joint infection (PJI) is a devastating complication after THA and is among the most common indications for hip revision arthroplasty (5). Although the rate of PJI after THA is relatively small (range 0.3%–2.2%), up to 16% of the THA revisions are performed due to infection (5). While the presence of PJI may be obvious in some patients, it more commonly presents with less specific manifestations such as unexplained pain (6). Synovial fluid aspiration is the cornerstone for PJI detection and treatment planning in such cases (7). Hip aspiration, however, is not always straightforward, as “dry tap” is a frequent occurrence, with a reported incidence rate of 23%–45% (8, 9).

Hip aspiration has been traditionally performed in the operating room (OR) by the orthopedic surgeon. More recently, ultrasound (US)-guided aspiration of the hip by an interventional radiologist has become an accepted approach to maximize the chance of a productive aspiration (10–12). Although this technique significantly reduces the cost and OR burden, it is not widely used in some countries and arthroplasty centers, where aspirations continue to be performed in the OR (13–16). In this setting, each dry tap results in a significant waste of OR staff, time, and resources. It also exposes the patient to an unnecessary invasive procedure with its potential morbidities and may delay their diagnosis and treatment. Predicting dry taps could enable the care providers to avoid such drawbacks.

In this study, we aimed to evaluate the accuracy of outpatient hip US in the prediction of dry tap in a series of patients suspected of hip PJI. We hypothesized that the absence of fluid collection in the US could predict the occurrence of dry tap in the OR.

## Patients & methods

This study was approved by the ethics review board of our institute, Moheb-Mehr Hospital, Tehran, Iran. Patients provided written consent before participation in the study. Between August 2019 and December 2021, patients indicated for hip revision arthroplasty that were suspicious to PJI due to clinical or lab data were prospectively enrolled in the study. Patients with a definite PJI due to the presence of a draining sinus (*n* = 5), patients with local conditions hindering a reliable US examination such as significant heterotopic ossification (*n* = 2), those with a cement spacer after a first-stage hip revision (*n* = 6), and patients with superficial infection precluding a safe aspiration (*n* = 1) were excluded from the study. Finally, 53 hips were recruited out of which 50 (47 patients) completed the study and were included. They consisted of 37 (78.7%) males and 10 (21.3%) females with a mean age of 55.2 ± 16.1 (range 28–80). Out of the 50 hips, 41 had a primary THA prosthesis and 9 had revision components.

Outpatient two-dimensional US of the hip was requested for all patients before performing hip aspiration. The radiologist was asked to report the presence of fluid collection and its exact location in/around the hip joint. The presence of any intra-articular or periprosthetic fluid accumulation whether as an anechoic area or debris contained, with increased through-transmission was defined as a fluid collection. Otherwise, it was regarded as a negative US investigation for fluid collection. US evaluations were performed by 15 different radiologists as determined by the patients’ preferences and their insurance regulations.

All patients underwent hip aspiration within one week after US evaluation. In the OR and under monitored anesthesia care, the patients were placed in a supine position. Under the guidance of the fluoroscope, hip aspiration was done using a 22G × 5” spinal needle or a nephrostomy access needle (18 gauge, 20 cm cannula) through anterior and/or lateral approaches. For this purpose, the needle was directed toward the medial and lateral neck of the prosthesis. If the aspiration was negative with this technique, the needle would then be advanced past the superolateral aspect of the neck of the prosthesis into the dependent portion of the joint, as previously described as the most reliable technique (17). In cases with a positive fluid collection in the US, the reported location was also specifically approached. Successful aspiration was defined as the ability to extract ≥1 ml of non-blood fluid. Aspiration of fewer than one ml was labeled as positive only if its culture would come back positive with bacteriology consistent with the result of the later intraoperative tissue culture, sent during the revision surgery. Saline instillation was not performed for dry taps. All aspirations were done under the supervision of the senior author (MA).

The synovial fluid samples were collected in different tubes containing BD BACTEC-PEDS-PLUS/F Medium (Becton Dickinson, Heidelberg, Germany), anaerobic blood agar, and Sabouraud agar for fungal culture. A separate sample was also sent for white blood cell count. In select cases, samples were also collected for Tuberculosis culture and Polymerase Chain Reaction study. Whenever enough fluid was not available for all culture media, the aerobic culture was given priority. The culture media were maintained in the incubator for up to 15 days for bacterial cultures and a minimum of 30 days for fungal cultures.

All patients underwent a single-stage or a two-stage revision surgery, based on their status according to the 2018 Musculoskeletal Infection Society (MSIS) criteria for PJI diagnosis (18). Intraoperatively during the later revision surgery, a total of five tissue samples were obtained from each hip. The results of intraoperative tissue cultures were regarded as the reference control for determining the success of fluid aspiration if the aspiration volume was ≤1 ml.

## Statistical analysis

According to the study by Ong et al. (19), the prevalence of dry tap in hip aspiration was 35.7%. According to this prevalence, type I error of 5%, and power of 80%, a number of 50 hips were identified enough for the evaluation of US sensitivity in the prediction of dry tap (20).

SPSS for Windows, version 16 (SPSS Inc., Chicago, Ill., USA) was used for statistical evaluations. Descriptive data were demonstrated with mean ± standard deviation or number and percentage, with 95% confidence interval (CI). Crosstab order was used to determine the sensitivity, specificity, positive predictive value (PPV), and negative predictive values (NPV) of US in the prediction of dry taps.

## Results

US evaluation revealed positive fluid collection in 58% (29 of 50) of the hips and was negative in 42% (21 of 50). Aspiration was successful in 46% (23 of 50) of the hips and was dry in 54% (27 of 50). Matching the results of US evaluation with aspiration showed that only 4.7% (one of 21) of the hips with the negative US for fluid collection had a successful aspiration (Table 1). This patient had chronic PJI and was previously treated with multiple debridement procedures and periods of intravenous antibiotic therapy. The aspirated fluid was less than one milliliter, but its culture turned positive for an uncommon type of Streptococcus (Streptococcus gordonii), consistent with the tissue culture results obtained during the revision surgery.

**Table 1:** Ultrasound ^a^ aspiration cross-tabulation.

		Aspiration result		Total
		Dry tap	Successful tap	
US evaluation	-ve for fluid collection	20	1	21
	+ve for fluid collection	7	22	29
Total		27	23	50

^a^ US, ultrasound.

75.9% (22 of 29) of the hips with a fluid collection reported by the US had a successful aspiration, too. Of the hips with positive US and negative aspiration, 71.4% (five of seven) were finally diagnosed to be infected. Evaluation of the accuracy of the US in the prediction of dry taps revealed a sensitivity of 95.7% (95% CI 69.8–99.8) and a specificity of 74.1% (95% CI 52.8–91.8). The PPV and NPV of the US in the prediction of aspiration success were 75.9% (95% CI 50.9–91.3) and 95.2% (95% CI 71.3–99.8), respectively. The Needed Number to Treat (NNT) to prevent one dry tap was 2.38. These results are summarized in Table 2.

**Table 2:** Accuracy of outpatient ultrasound for predicting aspiration results.

Index	Sensitivity	Specificity	Positive predictive value	Negative predictive value
Value	95.7%	74.1%	75.9%	95.2%
95% CI	69.8–99.8%	52.8–91.8%	50.9–91.3%	71.3–99.8

The 24.1% negative aspiration rate under the guidance of fluoroscopy when fluid was detected in the hip by US is a much higher figure than only 3% negative aspiration rate reported by Biliant et al. (21), in hips undergoing US-guided hip aspiration. Therefore, hip aspiration under US guidance in the radiology department is more accurate than aspiration in the OR under fluoroscopy guidance.

The mean OR time for a hip aspiration patient, from the time the patient entered the OR to the time the next patient entered, was 40.4 ± 18.2 min (95% CI 35.4–45.4). The mean number of C-arm shots per procedure was 3.8 ± 3.1 (95% CI 2.9–4.7). This figure was higher for the patients with a dry tap at 5.7 ± 5.1 (95% CI 4.3–7.1) [*P* value = 0.045, mean difference 1.9 (95% CI 0.04–3.76)].

Intraoperative tissue cultures were positive in 72% (36 of 50) of the hips, including *Staphylococcus aureus* (*n* = 9), *Staphylococcus epidermis* (*n* = 7), *E.Coli* (*n* = 5), *Klebsiella pneumonia* (*n* = 3), *Pseudomonas aeruginosa* (*n* = 3), *Proteous vulgaris* (*n* = 1), *Morganella morganii* (*n* = 1), *Enterococcus* (*n* = 1), *Citrobacter* (*n* = 1), *Streptococcus gordonii* (*n* = 1), and *Candida albicans* (*n* = 2). In two hips, the infection was polymicrobial.

## Discussion

We found that outpatient pre-aspiration US in the OR could accurately predict the chance of dry tap occurrence in THA patients suspected of PJI. Ultrasound predicted dry tap for twenty-one out of 50 hips, out of which actual dry tap was observed in 20 hips during aspiration (concordance rate of 95.2% on dry tap). Therefore, hip US before aspiration in the operating room can fundamentally decrease the rate of dry taps, which will minimize the undue exposure of the patients to an invasive procedure and unnecessary costs to the healthcare system, and expedite the treatment process. However, the US was not as accurate in predicting if a successful tap would follow a positive collection sign, and only 75.9% of the cases with the positive US led to successful aspiration.

Confirming a hip PJI diagnosis is often challenging and a dry tap aspiration further complicates this process (22). The study by Christensen et al. revealed that almost one-third of patients suspected of PJI have a dry hip aspiration, adversely affecting the process of PJI management (23). Various attempts to get a meaningful culture from joints with an initial dry tap have been suggested (10, 24), the most popular being the saline lavage technique. However, there is no consensus about the effectiveness of this approach. While some authors have reported increased sensitivity of PJI diagnosis following saline lavage and re-aspiration (24, 25), others have reported low sensitivity of such aspirates for PJI detection (11, 26). In addition, other parameters of aspiration fluid analysis, such as white cell count, neutrophil differentiation, and alpha defensin, cannot be relied on when lavage fluid is being analyzed (26). Of note, the 2018 International Consensus Meeting on Musculoskeletal Infection recommended against performing a saline lavage on dry tap occasions (18). Therefore, a dry tap is almost equal to a failed attempt, time and resource wasting, in spite of the hazardous exposure of the patient to an invasive procedure and ionizing radiation.

Attempts have been made to predict hip dry taps. Ong et al. (19) investigated the predictive role of various factors, including the number of prior hip surgeries, size of the femoral head, ESR/CRP values, and body mass index on the “dry tap” occurrence in 336 patients with suspected PJI following THA. Dry tap occurred in 35.7% of their patients. However, none of the evaluated factors were found to be predictive.

Ultrasound is an effective imaging modality for facilitating fluid aspiration by guiding the needle placement for arthrocentesis. Wei et al. (27) evaluated the efficiency of US in differentiating hip PJI (*n* = 50) from aseptic loosening (*n* = 12). Their results showed a PPV of 92.1% in the differential diagnosis of PJI and aseptic loosening. Another study revealed the high US efficiency in the evaluation of periprosthetic complications in THA patients by showing a perfect concordance between the US and MRI for detecting fluid collection around hip prostheses (28). Rendelli et al. (29) compared the sensitivity and specificity of fluoroscopy-guided- vs. US-guided joint aspirations performed at the radiology department in 52 hips suspicious of PJI. They found a sensitivity and specificity of 89% and 94% for US-guided joint aspirations in the detection of PJI, while the sensitivity and specificity of fluoroscopic joint aspiration were 60% and 81%, respectively. A similar study by Battaglia et al. (30) showed better sensitivity and specificity of US-guided aspiration than fluoroscopic-guided aspiration (69% vs. 27% and 94% vs. 75%, respectively).

Although many investigations acknowledge the superiority of US-guided hip aspiration, this procedure continues to be performed in the operating room by many surgeons all around the world (13–16). The reasons for adopting this policy may include inadequate interdisciplinary collaboration between surgeons and radiologists, fear of inadequate aseptic measures in the radiology department and suboptimal handling of the samples after aspiration, or simply unawareness of the accuracy of US-guided aspiration. We observed a high reliability of outpatient US in the prediction of dry tap, demonstrated by a very small rate of false negative. Although pre-aspiration US was less accurate in the prediction of successful taps, for interventional procedure such as hip aspiration, smaller false negatives are crucial and more important than a small false positive rate. High sensitivity (95.7%) of pre-aspiration US in the present study confirms its small false negative rate. These results could be informative for surgeons who perform hip aspiration in the OR, revealing that outpatient US evaluation is a reliable method to predict dry taps.

Although a preoperative US can help clinicians avoid a dry tap in the OR, only about ¾ of the cases with a positive fluid sign in the US will have a positive tap in the OR under fluoroscopy guidance. This is a lower figure compared to the 97% success rate reported by Biliant, when the aspiration takes place under the US control in the radiology department. Of note, most cases with the positive US and a dry tap were found to be infected in the current study, highlighting the significance of this limitation of fluoroscopy-guided aspiration. We believe that this significant drawback of performing hip aspiration in the operating room, as opposed to US-guided aspiration in the radiology department, merits consideration and suggests a potential need for a policy revision in centers where the former practice is in place.

Not only the outpatient US could help the surgeons avoid unnecessary aspiration procedures and their potential morbidities such as bleeding, nerve injury, and introducing a new infection to the joint (31), but also it reduces the financial and time burdens, both for patients and the health-care system. The average time from entering a patient to the OR for hip aspiration to the end of operation and preparation of the OR for the next patient was almost 40 min. Considering an average cost of about $37 per minute for running OR (32), avoiding one single dry tap aspiration could save around $1,480, excluding the other costs of the procedure. The reimbursement fee for an outpatient hip US study could be from $48 to $245 in different outpatient settings across the United States (33). Assuming an average cost of $146.5, and given the NNT of 2.38, for preventing a minimum cost of $1,480 for each dry tap, $348.67 would be spent on preoperative outpatient US evaluations, a clearly cost-effective figure. In addition, we exposed the dry tap patients to an average of 5.7 C-arm shots that could have been avoided by a prior US investigation.

To our knowledge, this is the first study assessing the predictive value of the pre-aspiration US study for dry tap occurrence. The prospective nature of the study adds to the reliability of the findings. However, it has some limitations, too. Fifteen different operators with different experience levels did US evaluations. However, this heterogeneity could also be regarded as a strength of the study because it better reflects real-world circumstances. A full cost analysis was not performed due to the lack of some necessary pieces of information. In addition, the measured OR time for hip aspirations could be institution-specific and not generalizable to all centers. Nonetheless, the limited calculations performed on the direct financial wastes of a dry tap reveal the high value of avoiding it, a fact that is unlikely to be fundamentally different for other centers.

In conclusion, performing outpatient US before hip aspiration in patients suspected of PJI following THA can accurately predict dry-tap aspirations, thereby decreasing the waste of time and resources in the OR, preventing patients’ exposure to ionizing radiation, and minimizing the delay in the diagnosis and treatment of PJI. Therefore, we suggest the adjunction of outpatient US to hip aspiration surgery in centers where this procedure is preferred to be performed in the OR. In such centers, we recommend repeating the US investigation in cases with a negative US study, and if still negative, planning for a tissue sampling. In the broader context, our findings support US-guided aspiration within the radiology department over x-ray-guided aspiration in the operating room, even when factoring in the benefits of a pre-aspiration ultrasound assessment.

## Data Availability

The raw data supporting the conclusions of this article will be made available by the authors, without undue reservation.
